# Prevalence of Peri-Implantitis: A Multi-Centered Cross-Sectional Study on 248 Patients

**DOI:** 10.3390/dj8030080

**Published:** 2020-08-03

**Authors:** Tommaso Weinstein, Tommaso Clauser, Massimo Del Fabbro, Matteo Deflorian, Andrea Parenti, Silvio Taschieri, Tiziano Testori, Luca Francetti

**Affiliations:** 1 Humanitas Dental Center, Humanitas Research Hospital, Rozzano, 20089 Milan, Italy; tommasoweinstein@me.com; 2 Department of Biomedical, Surgical, and Dental Sciences, University of Milan, 20122 Milan, Italy; tommaso.clauser@gmail.com (T.C.); silvio.taschieri@unimi.it (S.T.); info@tiziano-testori.it (T.T.); luca.francetti@unimi.it (L.F.); 3 IRCCS Orthopedic Institute Galeazzi, 20161 Milan, Italy; deflon@hotmail.it (M.D.); andrea@studioandreaparenti.it (A.P.); 4 Department of Periodontics and Oral Medicine, School of Dentistry, The University of Michigan, Ann Arbor, MI 48109, USA; 5 Private Practice, 22100 Como, Italy

**Keywords:** dental implant, peri-implantitis, implant supportive therapy, implant success

## Abstract

The aim of this multicenter cross-sectional study was to determine the prevalence of peri-implantitis and to assess its association with several patient- and implant-related factors. Patients with at least one implant, who came for a recall visit to one of the four centers over a period of five months, were enrolled. Presence of peri-implantitis (defined as bleeding on probing, exudate/suppuration, bone loss > 0.2 mm/year and increased pocket depth) and several other variables (e.g., smoking habits, history of periodontitis, diabetes) were recorded. Out of 248 enrolled patients (1162 implants), 10 patients had at least one implant with peri-implantitis (4.03%); a total of 14 implants were affected (1.20%). A statistically significant association between peri-implantitis and diabetes was found (OR 8.65; CI: 1.94–38.57). Smoking more than 10 cigarettes per day (OR: 0.53; CI 0.03–9.45) and history of periodontitis (OR: 2.42; CI: 0.49–11.89) were not found to be statistically associated with peri-implantitis. Even if implant therapy is a consolidated treatment, biological complications do happen. Strict supportive therapy recalls could lead to lower rates of peri-implantitis and earlier diagnosis.

## 1. Introduction

Implant therapy has dramatically changed dental rehabilitation in the last 40 years. A literature review, including only longitudinal studies with a 10-year follow-up, reported an implant survival of 94.6%, with a mean marginal bone loss of 1.3 mm [1]. Another systematic review confirmed how this therapy improved over the years: the survival rate at five-years of follow-up was 93.5% in studies published before 2000, while in the subsequent studies it was 97.1% [2].

However, implant therapy is not free of risks: in recent years, a steady increase in biologic complications like mucositis and peri-implantitis has been observed, which may influence the decisional trend among clinicians about saving compromised dentition or not.

Mucositis is defined as the reversible inflammation of peri-implant soft tissues. Conversely, peri-implantitis is characterized by the irreversible loss of bone support further than the physiological crestal bone remodeling. Peri-implantitis is defined, in accordance with the 2017 World Workshop on the Classification of Periodontal and Peri-Implant Diseases and Conditions, as: “a plaque-associated pathological condition occurring in tissues around dental implants, characterized by inflammation in the peri-implant mucosa and subsequent progressive loss of supporting bone” [3].

During past years, there was a lack of standardization of the case definitions, etiology and diagnostic criteria, leading to a wide range of prevalence of peri-implantitis [4]. The prevalence of peri-implantitis was 1 to 7% in accordance with a recent systematic review, and a subsequent meta-analysis found the prevalence of peri-implantitis at 22% [5].

Peri-implantitis is a serious multifactorial disease whose onset and progression rate may depend on patient, surgical and prosthesis-related factors, which could lead to the loss of the fixture. Up to date, there are no clear guidelines to treat it: as stated by Heitz-Mayfield et al., the outcome of the various treatments proposed is encouraging, but data are still too heterogeneous to draw any conclusion [6]. Certainly, in analogy with periodontal diseases, preventive measures can play a role in controlling the incidence of such a condition in the population. Current guidelines for the maintenance of implant restorations are poorly defined and often based on empiricism or traditional protocols for patients with natural dentition, rather than indicating what is most suitable for maintenance of implant-retained restorations and supporting tissues. Therefore, professional and home care maintenance guidelines are necessary for patients with tooth and implant-borne removable and fixed restorations to improve the health of supporting tissues, limit disease processes such as caries, periodontitis or peri-implant disease and improve the expected longevity of restorations, as well as the supporting teeth and implants themselves. In view of the increasing number of reports suggesting the importance of peri-implant maintenance programs, sound evidence-based guidelines are needed to provide direction to the dental health care provider, with the goal of improving the clinical outcomes for the patient, and reducing the incidence of peri-implant diseases.

Peri-implantitis stands as a great challenge for clinicians and researchers: understanding and describing the problem are the first steps in order to prevent and treat this disease.

This study aims to (1) determine the prevalence of the peri-implantitis in a multi-center cross- sectional study and (2) to assess its association with some patient-related and implant-related factors.

## 2. Materials and Methods

### 2.1. Study Design

This multi-center cross-sectional study involved four centers, all run by experienced clinicians, who shared the same guidelines and background in implant therapy.

All subjects signed an informed consent form before being included in the study. The study was conducted in accordance with the Declaration of Helsinki, and the protocol for this cross-sectional study was approved by the Institutional Review Board of the IRCCS Orthopedic Institute Galeazzi of Milan, Italy (Project identification code 2552377-L2058, Prot. Dsc 75/2019, June 18, 2019).

The objective of the study was to determine the prevalence of peri-implantitis.

An implant was considered affected by peri-implantitis when the following three conditions were fulfilled at the same time:

1. Bleeding on probing and/or presence of exudate/suppuration;
2. Peri-implant bone loss greater than 0.2 mm/year, according to Albrektsson et al. 1986 [7];
3. ≥1 mm pocket depth compared with previous examinations.

### 2.2. Study Population

The observational period lasted 5 months. Patients with at least one dental implant restored and in function for at least 12 months, presenting in this period for dental hygiene recall, were enrolled. Patients unable to undergo radiographical examination were excluded.

### 2.3. Clinical Examination

Data were collected through a specific form.

Specifically, the following information was recorded:

- Systemic risk factors (smoking, history of periodontitis and diabetes);
- Implant characteristics and manufacturer;
- Date of implant insertion and loading;
- X-ray at prosthetic loading;
- Keratinized tissue;
- Probing depth assessed at 6 aspects around each implant;
- Recession, defined as implant neck exposure or worst;
- Plaque index;
- Bleeding on probing;
- X-ray where required (once a year or in case of bleeding on probing associated with increased probing);
- Recall visit dates;
- Type of retention of the prosthesis (cemented/screwed).

Data were collected before any professional dental hygiene procedure performed by a dental hygienist. There were two possible scenarios.

**Figure 1:**
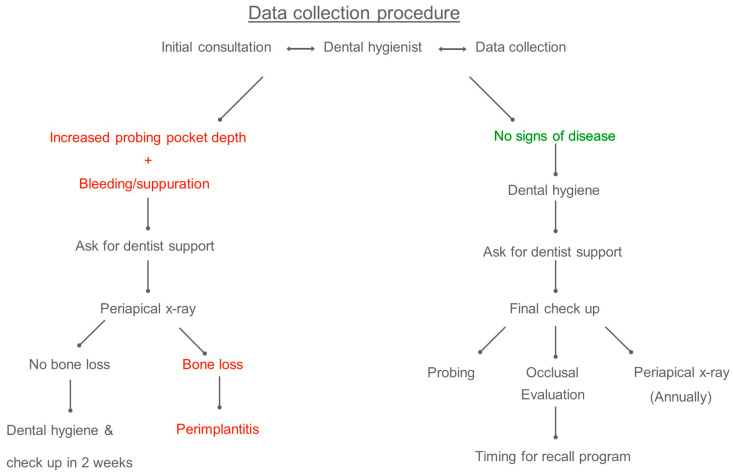
Workflow with the two possible scenarios: 1. no signs of disease (**right side**); 2. bleeding or suppuration on probing associated with increased pocket depth (**left side**).

1. If there were no signs of disease, the hygienist carried out the dental hygiene procedure, involving the use of ultrasonic devices, titanium scalers, air-flow powder and polishing. The dental hygienist later called the dentist for a final check-up evaluation. The dentist performed a complete clinical inspection. Every implant was probed again and occlusal evaluation was performed with 40µ articulation paper (occlusal contact required) (Arti-check, Bausch) and with 8µ (Hanel Shimstock Foil, Coltene) (no contact needed). Occlusal adjustment was performed when needed. Peri-apical X-rays of the implants were taken annually. At the end of the procedure, the timing for recall appointment was decided.
2. If the dental hygienist registered bleeding or suppuration on probing with increased pocket depth, he/she asked for dentist support. The dentist took a peri-apical X-ray of the implant(s) involved: if there was greater than expected bone loss with respect to the last periapical X-ray, the implant was considered affected by peri-implantitis and the relative treatment plan was discussed. Otherwise, the dental hygiene was performed, and a follow-up visit was scheduled in 2 weeks. (see Figure 1).

### 2.4. Statistical Analysis

Data were analyzed using Microsoft Excel and the R software (package fmsb). Prevalence is reported at implant level. Odds ratio (OR) and 95% confidence interval (CI) calculations were performed using the patient as a statistical unit (*n* = patient); null hypothesis is OR = 1.

## 3. Results

Two hundred and forty-eight patients were included (58.5% women, 41.5% men); mean age was 63.4 years for women and 62.5 for men (range between 20.7 and 92.4 years); a total of 1162 implants were inserted (91.73% Biomet 3i, Palm Beach, FL, USA). Non-smokers were 87.29%, 6.15% smoked less than 10 cigarettes/day and 6.56% smoked more than 10 cigarettes/day. Patients affected by diabetes type I were 6.48%, while patients with a history of periodontitis were 59.84%. The frequency of recall visit was three months (40 patients, 233 implants), four months (66 patients 370 implants) and six months (136 patients, 543 implants) (not registered 6 patients and 16 implants).

Regarding superstructure, 828 implants were cemented while 334 were screw-retained.

**Figure 2:**
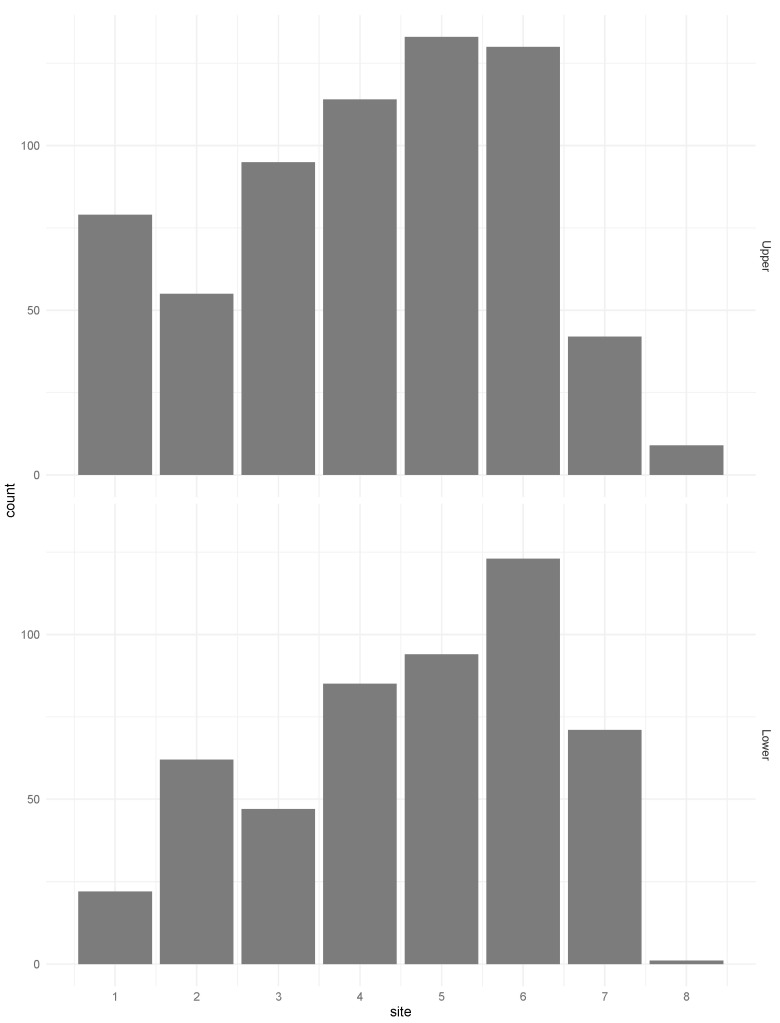
Distribution of implant sites for upper jaw and lower jaw.

Other study population characteristics are illustrated in Figure 2.

The implants diagnosed with peri-implantitis were 14 (1.20% of implants). Specifically, in implants with a follow-up duration lower than eight years, peri-implantitis was recorded in 0.86% of the cases, while in implants with more than eight years of follow-up, the incidence of peri-implantitis was 2.02%. At a patient level, peri-implantitis occurred in 10 patients (six females, four males; 4.03% of the total patients).

Cement-retained prostheses were found in 100% of the cases affected by peri-implantitis.

**Figure 3:**
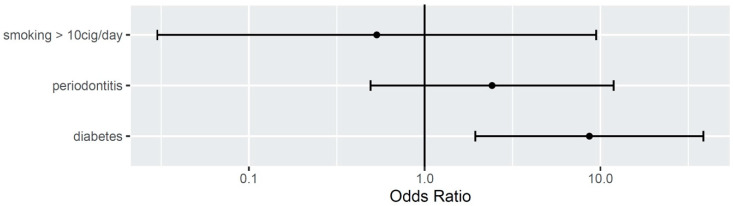
Odds ratio for smoking, periodontitis and diabetes.

Smoking more than 10 cigarettes a day (OR 0.53; CI: 0.03–9.45) and history of periodontitis (OR: 2.42; CI: 0.49–11.89) did not show as significant risks factors for peri-implantitis in this sample. Diabetes was significantly associated with peri-implantitis (OR 8.65; CI: 1.94–38.57) (see Figure 3).

Eight implants affected by peri-implantitis where then treated with a non-surgical approach, and one implant was treated with open flap scaling. These nine implants were still in place at the last follow-up visit. One implant was removed. Four implants where lost to follow-up.

## 4. Discussion

The wide spreading of dental implants started in the late 1990s, rapidly becoming one of the most used techniques in dentistry. Dental implants allow to treat edentulism with fixed rehabilitations capable to improve the quality of life of the patients in terms of function and esthetics.

Unfortunately, implant therapy may present adverse events. Technical complications, affecting mechanical components and superstructures, were identified in a Consensus Statement in 2018 [8] as: (1) fracture of abutments, (2) fracture of abutments screws, (3) fracture of occlusal screws, (4) abutment or screw loosening, (5) ceramic fractures or chipping and (6) loss of retention.

Mucositis and peri-implantitis are the main biological complications affecting hard and soft peri-implant tissues. From a microbiological point of view, there are similarities between these two diseases. In fact, a study by Zhuang et al. [9] found that periodontal pathogens are found in periodontal and implant sites. Specifically, Porphyromonas Gingivalis and Fusobacterium nucleatum were significantly associated only with periodontitis, while Aggregatibacter actinomycetemcomitans was associated with both periodontitis and peri-implantitis. These diseases share similar physiopathological patterns with gingivitis and periodontitis. Development of peri-implantitis is generally faster than periodontal disease, mainly because tissues around implants are less vascularized than around teeth, and hence the immune response to a microbial challenge is less effective. Prevention through regular maintenance programs of professional oral hygiene is the best way to treat peri-implantitis, together with immediate intervention when necessary.

Literature about peri-implantitis has increased in recent years, together with the widespread use of dental implants. A recent systematic review and meta-analysis found the prevalence of peri-implantitis at the implant level ranging from 1.1% to 85.0%, and the incidence from 0.4% within three years to 43.9% within five years [10]. It is interesting to highlight the wide range described. The authors stated in the conclusion that the studies included were extremely heterogeneous, with different diagnostic criteria. These results are in accordance with the finding of a research with a similar purpose, carried out by Derks in 2015 [5]. Some of the main issues were the definitions of clear thresholds to properly identify a case of peri-implantitis. Derks et al. [5] in their research included 12 studies and found 8 different thresholds, in terms of marginal bone loss amount.

Another paper evaluated how the prevalence of peri-implantitis changed in the same population, changing the criteria to define it [11]. When using a probing pocket depth (PPD) > 4 mm and BOP and bone loss > 2 mm as diagnostic criteria for peri-implantitis, the prevalence was 47%. If the threshold changed to PPD > 6 mm and BOP and bone loss > 3 mm, the prevalence decreased to 11%.

The findings of the present study revealed a prevalence of peri-implantitis of 1.20% at the implant level and 4.03% at the patient level. At present, it is difficult to compare these results with the others in the literature, due to the different criteria applied.

The diagnosis of peri-implantitis could be made only with the correlation of different clinical and radiographic parameters which should be recorded periodically. It is important to determine the baseline in terms of marginal bone level through a peri-apical X-ray and probing pocket depth. The latter can vary considerably, depending on the peculiar macro-geometry of the single dental implant system features, and the subsequent effects in the development of the biological width. Further, the experience of the operator and the force applied to the probe can play a role. In the present study, any changes ≥ 1 mm in probing depth from the baseline associated with bleeding on probing and/or presence of exudate/suppuration called for a peri-apical X-ray. If bone loss > 2 mm was detected, a diagnosis of peri-implantitis was made.

It does not exist a single diagnostic criterion to determine peri-implantitis, and the clinician should probe during every recall appointment, evaluate any changes and the health of the peri-implant tissues and take X-rays every year to control the stability or marginal bone. In this study, peri-implantitis was defined as the combination of bleeding on probing > 0 and/or the presence of exudate/suppuration, peri-implant bone loss greater than expected in accordance with the criteria proposed by Albrektsson et al. in 1986 [7] and increased pocket depth compared with previous examinations. These criteria are in accordance with the findings of the 2017 World Workshop on the Classification of Periodontal and Peri-Implant Diseases and Conditions [3] and 2019 FDI Peri-implant Diseases Project: Consensus reports from the FDI workshop on prevention, diagnosis and treatment [12]. Hopefully, in the very near future, by applying these criteria, more homogeneous studies will be performed, and data could be compared in a profitable way.

All the cases affected by peri-implantitis showed a cement-retained restoration. In the literature, the benefit among cement- and screw-retained restorations is highly debated. Specifically, one of the problems with cement-retained restorations is the cement remnants which are strongly associated with the development of peri-implantitis [13], although there is a strong difference regarding the type of cement adopted, definitive versus provisional [14]. Specifically, authors compared the use of zinc oxide-eugenol cement and a methacrylate cement, founding that the latter was associated with development of suppuration. In the present study, zinc oxide-eugenol cement was used in every cement-retained restoration. Nevertheless, taking in account the high number of implants involved in this study suggests caution to draw definitive recommendations regarding this topic.

Despite the absence of specific evidence in the literature, it may be hypothesized that the high level of experience of the clinicians involved played an important role in such a valuable result. Nevertheless, probably the most important factor was the strict protocol of maintenance and the motivation given to the patients treated. In this paper, every patient attended almost twice a year to a dental hygiene. The frequency of recall visits was based on the risk factors of the patient and was re-scheduled every time, based on the level of home care. The dental hygiene was performed by a hygienist with the supervision of a dentist, usually the ones who performed the prosthetic treatment. This is a very important aspect because the clinician was aware of the clinical baseline of the patient and could quickly intercept any alteration of the treatment and perform the occlusal review.

Smoking, history of periodontitis and diabetes are well-recognized risk factors for developing peri-implantitis. Specifically, a recent consensus conference confirmed that patients with a history of severe periodontitis, poor plaque control and who failed to attend a regular recall program are more susceptible to develop peri-implantitis. Conversely, data about smoking and diabetes are inconclusive [3]. In this paper, the only risk factor with statistical significance is diabetes. Chronic hyperglycemia interferes at various levels on bone metabolism: despite that the literature suggests that implant therapy in diabetic patients can have high survival rates, a stringent glycemic control is strongly suggested. [15] Similarly, in spite, the use of tobacco seems not to affect implant therapy, though the effects on peri-implant tissues are quite evident and clinicians should encourage patients to quit this habit [16].

The influence of supportive therapy is well recognized as one of the main factors in the long-term prognosis of implant treatment.

Lin et al. recently published a systematic review and meta-analysis about the effects of supportive treatment on implant survival rate [17]. They included clinical studies evaluating groups of patients with and without supportive treatment after implant therapy, with at least one year of follow-up. The supportive treatment group significantly showed a higher survival rate (RR: 1.10; *p* < 0.001) and lower prevalence of peri-implantitis (RR: 0.25; *p* < 0.001) and peri-implant mucositis (RR: 0.57; *p* < 0.001) than the non-supportive treatment group.

Monje et al. found a 25% reduction in the incidence of peri-implantitis for patients with regular supportive treatment care, compared with those not receiving maintenance [18], and this tendency was confirmed by a recent systematic review [19].

Rösing et al. in a critical review underlined that replacing compromised teeth with dental implants does not avoid biological complications. Clinicians should be aware that the underlying genetics, microbiology, functional demands and behavioral habits associated with oral diseases are not necessarily modified with the placement of dental implants [20].

At present, protocols for implant therapy suitable for any patient are not well-defined, given the high variability encountered among different patients. A recent panel of experts stated that the maintenance care protocol must be individually determined, and a baseline condition identified for each patient. The recall frequency must have a specific periodicity that can vary from patient to patient according to the individual needs, and bone levels must be radiographically checked at least every two years, unless specific needs require a shorter interval [21].

## 5. Conclusions

Implant therapy is a very reliable treatment to restore function and esthetics in case of tooth loss. Nevertheless, biological complications such as peri-implantitis may occur and clinicians should advise patients that the treatment will not end with prosthetic loading. Supportive therapy is mandatory in order to properly prevent and intercept the inflammation around implants. Strict and individualized recall programs should be detailed for every implant patient.
